# Smoking cessation in the elderly as a sign of susceptibility to symptomatic COVID-19 reinfection in the United States

**DOI:** 10.3389/fpubh.2022.985494

**Published:** 2022-11-22

**Authors:** Wataru Ando, Takeshi Horii, Mitsuki Jimbo, Takayuki Uematsu, Koichiro Atsuda, Hideaki Hanaki, Katsuya Otori

**Affiliations:** ^1^Department of Clinical Pharmacy, Center for Clinical Pharmacy and Sciences, Kitasato University School of Pharmacy, Shirokane, Japan; ^2^Department of Pharmacy, Kitasato University Medical Center, Kitamoto, Japan; ^3^Laboratory of Pharmacy Practice and Science 1, Division of Clinical Pharmacy, Research and Education Center for Clinical Pharmacy, Kitasato University School of Pharmacy, Sagamihara, Japan; ^4^Biomedical Laboratory, Division of Biomedical Research, Kitasato University Medical Center, Kitamoto, Japan; ^5^Infection Control Research Center, Omura Satoshi Memorial Institute, Kitasato University, Shirokane, Japan

**Keywords:** COVID-19, smoking, reinfection, data analysis, database, United States

## Abstract

**Background:**

We aimed to clarify the relationship between coronavirus disease 2019 (COVID-19) reinfection and basic disease and smoking status.

**Methods:**

The electronic health records of 165,320 patients with COVID-19 from January 1, 2020, to August 27, 2021, were analyzed. Data on age, race, sex, smoking status (never, current, former), and basic disease were analyzed using Cox proportional hazard models.

**Results:**

In total, 6,133 patients (3.7%) were reinfected. The overall reinfection rate for never, current, and former smokers was 4.2, 3.5, and 5.7%, respectively. Although the risk of reinfection was highest among former smokers aged ≥65 years (7.7% [422/5,460]), the reinfection rate among current smokers aged ≥65 years was 6.2% (341/5,543). Among reinfected patients, the number of basic diseases was higher in former smokers (2.41 ± 1.16) than in current (2.28 ± 1.07, *P* = 0.07) and never smokers (2.07 ± 1.05, *P* < 0.001). Former smokers who are older may have been exposed to factors that increase their risk of symptomatic COVID-19 reinfection.

## Introduction

Coronavirus disease 2019 (COVID-19) continues to spread, with severe cases occurring worldwide. Chronic diseases, such as hypertension, diabetes, and obesity, are risk factors for severe COVID-19 (1, 2). It was important to understand these risks earlier when COVID-19 was identified; however, they were not fully elucidated. With the widespread availability of vaccines, the risk of reinfection has been reported to be ~1% or less (3).

A recent meta-analysis showed a lower reinfection rate among vaccinated individuals than among unvaccinated individuals (0.32 vs. 0.74%). However, the rate varied depending on the time of the epidemic, increasing to 3.31% during the Omicron epidemic (4). Factors that reduce the risk of reinfection include vaccines and a history of COVID-19, which greatly reduces the risk of reinfection (5). Additionally, the low likelihood of reinfection after infection is presumed to be equivalent to increased protection against infection by vaccination (6). However, the data on reinfection exclude effects related to smoking status.

This information regularly varies as the period of the COVID-19 epidemic lengthens. Although the vaccine is effective in older people (7), it is important to anticipate the risks associated with reinfection (8). There have been indications that reinfection may occur even in the presence of neutralizing antibodies.

The effects and protective factors associated with reinfection and basic diseases are not fully understood (9, 10). Flacco et al. (11) reported an increased risk of reinfection when risk factors include at least one of the following: diabetes, hypertension, major cardiovascular disease, chronic obstructive pulmonary disease (COPD), kidney disease, and cancer.

In contrast, smoking has been considered a risk factor since the beginning of the COVID-19 epidemic (12); however, there are conflicting reports on its impact on severe disease and reinfection. For example, de Lusignan et al. (13) reported that the positivity rate for severe acute respiratory syndrome coronavirus 2 (SARS-CoV-2) is higher in adult males and Black individuals. Simultaneously, smokers are less likely to test positive. Similarly, there are conflicting reports regarding reduced and worsening risk (14, 15). The effect of smoking on COVID-19 has been associated with increased mortality and disease severity. However, the causal relationship is yet to be determined, although the risk of infection appears to have reduced in current smokers compared with that in never smokers, and the risk of severity and death appears to have increased in former smokers (16). Interestingly, a higher degree of COVID-19 progression has been reported in smokers, particularly in younger smokers under the age of 45 (17). However, the risk of reinfection is yet to be evaluated. Therefore, using a large database, we aimed to examine the relationship between basic disease and patient background, including smoking status, with reinfection.

## Methods

### Study design and data source

The electronic health records (EHRs) used in this study were provided by the Healthjump database (Healthjump Inc., Philadelphia, PA, USA) and the COVID-19 Research Database Consortium (https://covid19researchdatabase.org). All personal information in this database is anonymized.

### Participants and definitions

The Healthjump database receives and anonymizes the origin of the EHR data of over 40 million unique patients and several healthcare organizations across the United States (18). EHR data from January 1, 2020, to August 27, 2021, were analyzed. Patients with SARS-CoV-2 RNA positivity (International Statistical Classification of Diseases and Related Health Problems, Tenth Revision; ICD-10: U07.1) aged ≥20 years were included. The patient exclusion criteria were as follows: those with missing information regarding age, sex, and diagnosis date of COVID-19; suspected SARS-CoV-2 positivity (ICD-10: U07.2); type 1 diabetes; and body mass index (BMI) ≤ 15 kg/m^2^ or > 50 kg/m^2^. Data obtained included age; sex; racial identity, including ethnicity (Hispanic or non-Hispanic white, non-Hispanic Black, Asian, and others or unknown); 1-year medication history of asthma; COPD before SARS-CoV-2 diagnosis; diagnosis of type 2 diabetes (T2D) (ICD-10: E11.65, E11.8, E11.9); hypertension (ICD-10: I10); hyperlipidemia (ICD-10: I78); cardiovascular disease, including angina pectoris, myocardial infarction, and other acute ischemic heart diseases (ICD-10: I20-I25); chronic asthma (ICD-10: J45); COPD (ICD-10: J44.9); interstitial pneumonia (IP) (ICD-10: J84); and BMI, measured by hospital staff or self-reported up to 6 months before the analysis. When more than one value was available for analysis, the value obtained closest to the diagnosis of COVID-19 was considered.

The Current Procedural Terminology 4th edition and the Healthcare Common Procedure Coding System codes were used for vaccination history. Patients with a code for vaccination services (Pfizer, 91300; Moderna, 91301; Johnson & Johnson, 91303) were defined as having received the vaccination, and patients without the code were defined as “not vaccinated” or “no vaccination history registered.” In addition, vaccination was defined as complete if a second code was present 18 days later.

If the diagnosis code of COVID-19 reappeared 90 days after the disappearance, it was defined as reinfection. Since reinfection is only identified by codes, reinfection was treated as symptomatic reinfection in this study. Therefore, data on reinfections that were not diagnosed and were asymptomatic were excluded.

Reinfection was considered the study outcome, while T2D, obesity, hypertension, hyperlipidemia, asthma, COPD, IP, and lung cancer history were evaluated. The number of days from the date of the first diagnosis to reinfection was calculated and used in a Cox proportional hazards model. When reinfection was not confirmed, the number of days between the initial infection and the end of the observation period of this study was used. Patients with BMI ≥30 kg/m^2^ were classified as obese (19).

Smoking was defined as occasional light to heavy smoking. Those using e-cigarettes and vapor cigarettes and those chewing tobacco were excluded. Smoking status was defined as current, former (history of smoking in the past), never, and unknown (without any information). The relationship between smoking status and the number of basic diseases that significantly affected reinfection was examined.

### Statistical analysis

Patient data that followed a normal distribution (age and BMI) are expressed as mean ± standard deviation values. The observation period is expressed as median values (interquarter range); continuous variables were analyzed using a one-tailed unpaired *t*-test. Categorical variables were analyzed using the χ^2^ test and are expressed as absolute numbers and/or percentages. For the analysis of reinfection and smoking status of the three groups, the Yates-corrected χ^2^ test was performed in each of the two groups, followed by a Bonferroni correction (indicated *P*-value was tripled). Following analysis of variance, the Tukey–Kramer test was used to compare continuous variables among the three groups. Hazard ratios (HRs) for reinfection risk were analyzed using a Cox proportional hazards model adjusted for sex, age (≥65 years), BMI (≤30 kg/m^2^), history of T2D (yes/no), hypertension (yes/no), hyperlipidemia (yes/no), cardiovascular disease (yes/no), asthma (yes/no), COPD (yes/no), IP (yes/no), lung cancer (yes/no), and smoking status (never, current, and former). In addition, the Cox proportional hazards assumption was tested. All statistical analyses were performed using STATA 16.0, Statistics for Windows (Stata Corp LLC, College Status, TX, USA), and a *P*-value of < 0.05 was considered statistically significant.

### Ethical considerations

This study was approved by the Ethical Committee of Kitasato University Hospital (No. 20-366). Since unlinked, anonymized data were used, the ethics committee confirmed that this study was not subject to compliance with the Ethical Guidelines for Medical and Health Research Involving Human Subjects, and the requirement for informed consent was waived.

## Results

The mean age of the 165,320 (72,994; 44.2% males) patients with COVID-19 was 51.0 ± 17.6 years. The number of reinfected patients was 6,133 (3.7%; mean age 51.0 ± 17.6 years). The median observation period was 242 (184–316) days for patients with only initial infection and 167 (122–230) days for those with reinfection. The interracial data and BMI are presented in Table 1. The number of initial and reinfected cases by region is shown in Supplementary Table S1.

**Table 1:** Characteristics of patients with COVID-19 during initial infection and reinfection.

	**Initial infection**	**Reinfection**	**Total**
Number of patients	159,187	6,133	165,320
Age, mean (SD), years	50.7 (17.6)	57.0 (16.6)	51.0 (17.6)
Sex male/female, *n* (%)	70,579 (44.3)/88,608 (55.7)	2,415 (39.4)/3,718 (60.6)	72,994 (44.2)/92,326 (55.8)
Non-Hispanic white, *n* (%)	50,132 (31.5)	2,276 (37.1)	52,408 (31.7)
Hispanic white, *n* (%)	27,752 (17.4)	745 (12.1)	28,497 (17.2)
Non-Hispanic black, *n* (%)	16,105 (10.1)	747 (12.2)	16,852 (10.2)
Asian, *n* (%)	1,113 (0.7)	37 (0.6)	1,150 (0.7)
Others and unknown, *n* (%)	64,085 (40.3)	2,328 (38)	66,413 (40.2)
BMI ≥30 kg/m^2^, *n* (%)	69,271 (43.5)	3,107 (50.7)	72,378 (43.8)
BMI, mean (SD), kg/m^2^	31.1 (9.4)	31.6 (10.6)	31.1 (9.4)
T2D, *n* (%)	29,575 (18.6)	1,610 (26.3)	31,185 (18.9)
HT, *n* (%)	61,977 (38.9)	3,367 (54.9)	65,344 (39.5)
HL, *n* (%)	63,067 (39.6)	3,548 (57.9)	66,615 (40.3)
CD, *n* (%)	14,772 (9.3)	1,007 (16.4)	15,779 (9.5)
Asthma, *n* (%)	6,149 (3.9)	471 (7.7)	6,620 (4)
COPD, *n* (%)	6,820 (4.3)	512 (8.3)	7,332 (4.4)
IP, *n* (%)	918 (0.6)	144 (2.3)	1,062 (0.6)
Lung cancer, *n* (%)	397 (0.2)	28 (0.5)	425 (0.3)
Current, *n* (%)	34,360 (21.6)	1,251 (20.4)	35,611 (21.5)
Former, *n* (%)	15,178 (9.5)	926 (15.1)	16,104 (9.7)
Never, *n* (%)	38,374 (24.1)	1,664 (27.1)	40,038 (24.2)
Observation period, median (IQR [25%−75%]), day	242 (184–316)	167 (122–230)	241 (178–312)

COVID-19, coronavirus disease; SD, standard deviation; BMI, body mass index; T2D, type 2 diabetes; HT, hypertension; HL, hyperlipidemia; CD, cardiovascular disease; COPD, chronic obstructive pulmonary disease; IQR, interquartile range.

**Figure 1:**
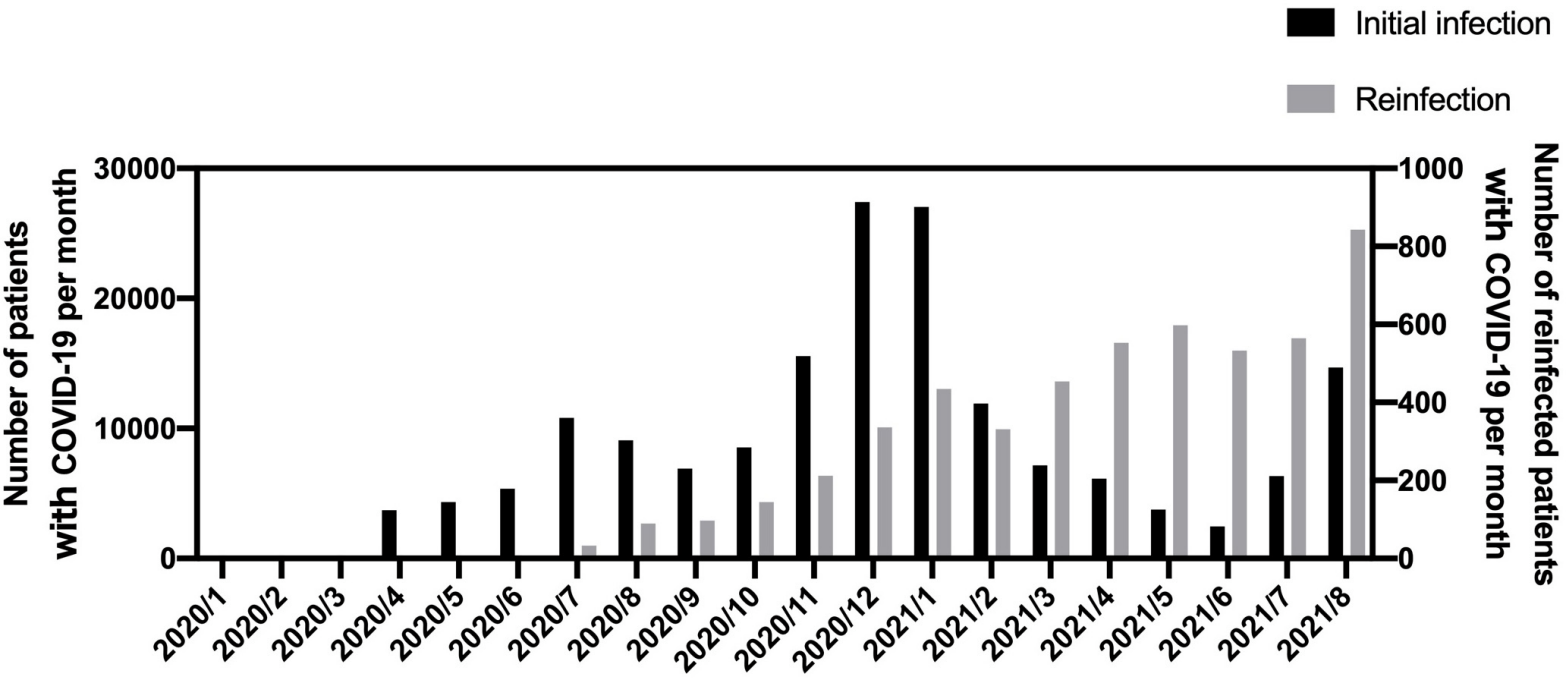
Number of infected and reinfected patients per month in this study. COVID-19, coronavirus disease 2019.

The number of infected patients peaked in December 2020 and January 2021 with 27,423 and 27,039 patients, respectively (Figure 1). Reinfections occurred around July 2020, with a reinfection rate of 0.36% in August 2020 (after 8 months of observation), which increased to 3.85% in August 2021. The reinfection rate was slightly lower among vaccinated patients who received two doses than those who received only one dose, although the data supporting these findings are limited (Supplementary Table S2).

**Figure 2:**
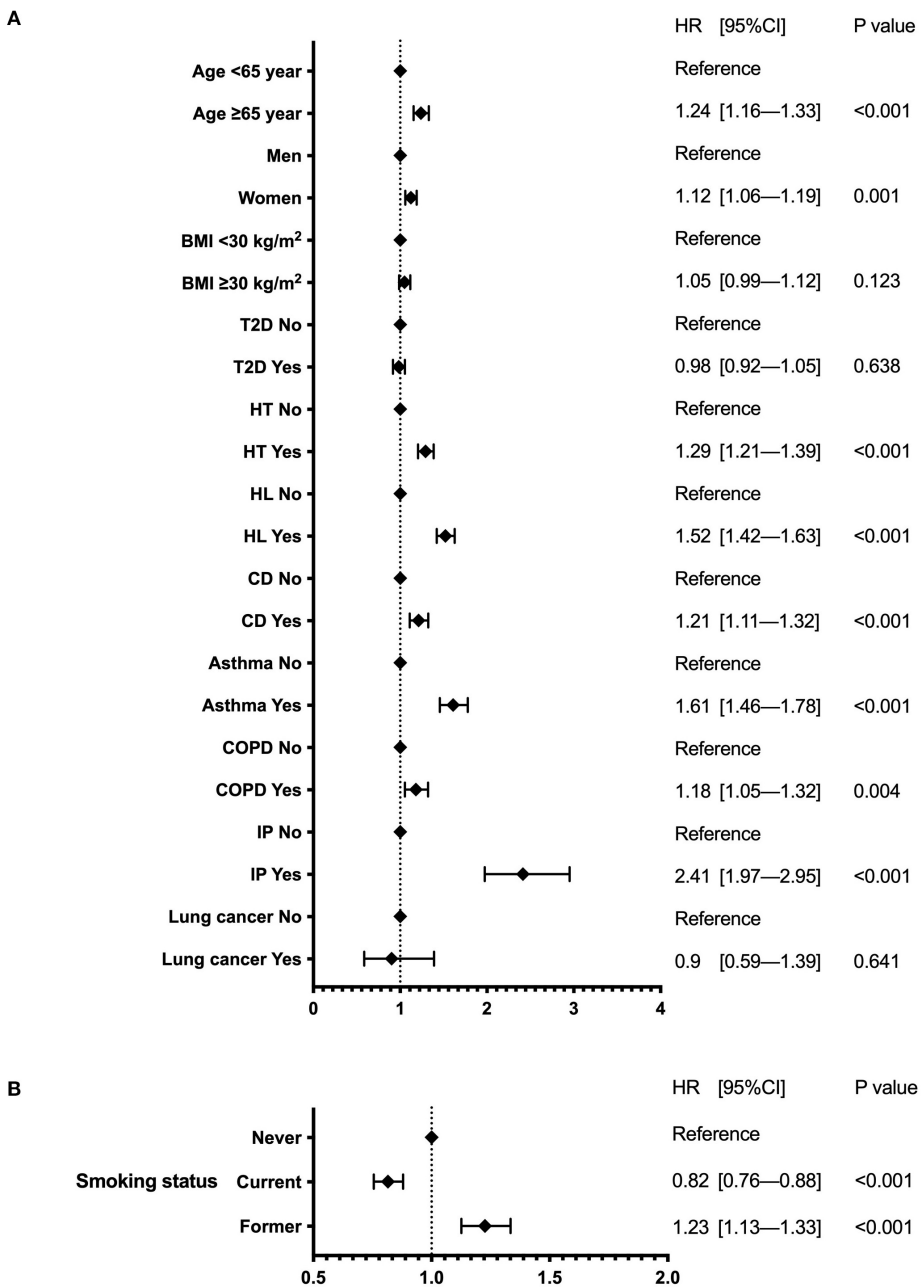
Forest plot of reinfection risk. The forest plot indicates the hazard ratios (diamonds) and 95% confidence intervals (horizontal bars) for reinfection risk. **(A)** Patient background and underlying disease. **(B)** Smoking status. BMI, body mass index; CI, confidence interval; HR, hazard ratio; T2D, type 2 diabetes; HT, hypertension; HL, hyperlipidemia; CD, cardiovascular disease; COPD, chronic obstructive pulmonary disease; IP, interstitial pneumonia.

Patients with IP had the highest risk of reinfection, followed by those with asthma and hyperlipidemia, with significantly higher HR (Figure 2A). The risk was also significantly higher in patients aged >65 years; women; and those with a history of hypertension, cardiovascular disease, and COPD. In contrast, obesity, T2D, and lung cancer showed no association. Regarding smoking status, current smokers had a significantly lower risk of reinfection than never smokers (HR 0.82 [0.76–0.88]; *P* < 0.001), while former smokers had an HR of 1.23 (1.13–1.33), *P* < 0.001 (Figure 2B).

**Figure 3:**
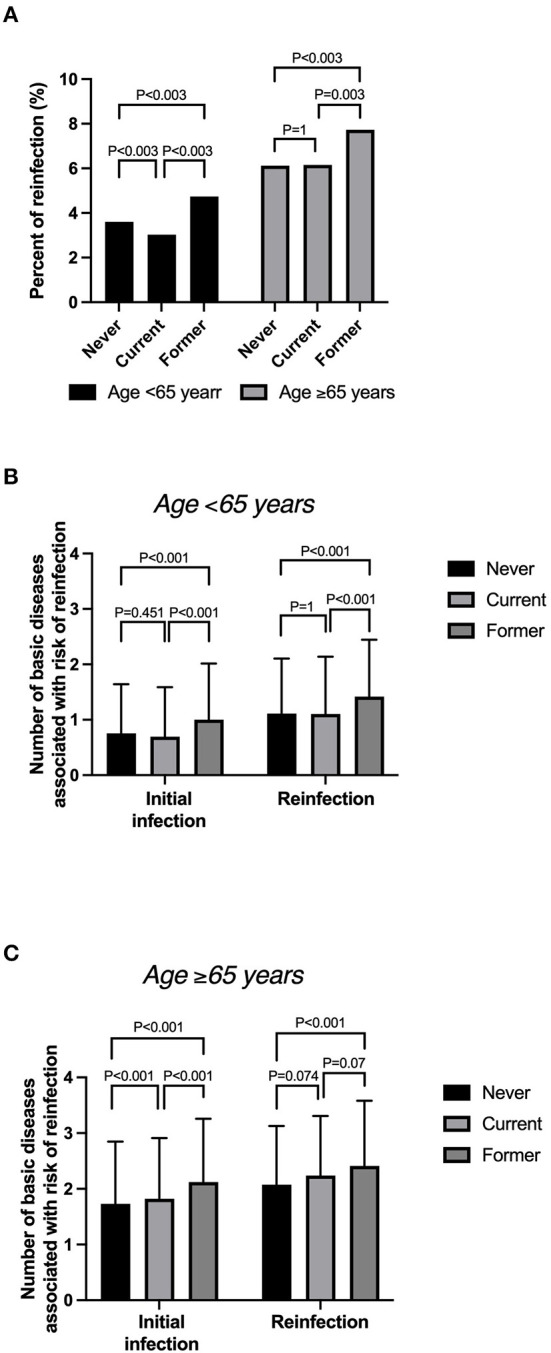
**(A)** Reinfection rate by smoking status among patients aged >65 and <65 years. **(B)** Mean number of basic diseases by smoking status among those aged <65 years. **(C)** Mean number of basic diseases by smoking status among those aged ≥65 years.

The overall reinfection rates by smoking status were 4.2% (1,664/40,049), 3.5% (1,251/35,612), and 5.7% (926/16,105) among never, current, and former smokers, respectively. The risk of reinfection was highest in former smokers aged ≥65 years: 7.7% (422/5,460) (Figure 3A). Current smokers aged ≥65 years had a reinfection rate of 6.2% (341/5,543). Regardless of the smoking status, the reinfection rate was higher among patients aged ≥65 years than among those younger.

For patients aged <65 years, the number of basic diseases associated with the risk of reinfection was <1 in patients with an initial infection but tended to be slightly higher among patients with reinfection. The number of basic diseases was significantly higher at the initial infection among patients aged <65 years (Figure 3B). In addition, the average number of basic diseases was >1 among these patients (Figure 3C). The number of basic diseases was significantly higher among former smokers than among never smokers. Furthermore, the number of reinfected patients was higher among former smokers than among current smokers aged ≥5 years.

## Discussion

This study showed that the risk of reinfection was higher among patients aged >65 years, and the highest rate of reinfection was observed among former smokers. Furthermore, former smokers who were older had a higher number of basic diseases, such as a higher risk of reinfection, than non-smokers and current smokers. The results suggest that former smokers were at potential risk of reinfection and that smoking cessation among older people was a sign of susceptibility to COVID-19 reinfection. In addition, this study reconfirmed that it is necessary to be cautious about reinfection if patients have many basic diseases. This study found that smoking affects the rate of symptomatic reinfections; however, results for non-symptomatic reinfections excluded from the database are unavailable. Therefore, this study's results do not represent the impact of smoking on all reinfections. The data were not obtained during the outbreak of the Omicron variant; however, the analysis provides important suggestions for future infectious disease control and prevention measures.

In a meta-analysis on smoking and COVID-19, the summary relative risk of death was 1.29 (95% CI 1.03–1.62) for current smokers and 1.25 (95% CI 1.11–1.42) for former smokers compared with never smokers. Although the risk of mortality increased for those who smoke or have smoked, no significant difference is observed in the risk of death across smoking status (20). Other meta-analyses have shown that the risk of severe disease is higher in patients with a smoking history (21).

Current smokers who were older had a 2-fold increase in the reinfection ratio compared with those aged <65 years (3 vs. 6%) in this study. This increased ratio was slightly higher than that of former and never smokers. The highest rate of reinfection was observed among former smokers who also had a higher number of basic diseases, which may have increased the risk of reinfection; this indicates that tobacco cessation does not directly increase the risk of reinfection.

A meta-analysis by Patanavanich et al. found that smoking increases the risk of COVID-19. In the findings on smoking and COVID-19, a meta-analysis of a total of 11,590 patients on smoking and COVID-19 in the early 2020 pandemic concluded that smoking is a risk factor for COVID-19 progression, with smokers having higher odds of COVID-19 progression than non-smokers (OR 1.91, 95% CI 1.42–2.59, *P* = 0.001) (22). However, other studies have shown that it lowers this risk. The complex issues of regional, national, and racial differences make it difficult to provide a definitive answer. The smoking rate among the patients with SARS-CoV-2-positive in this study was 39.0%, which is higher than the smoking rate of 7–13% in the United States (23). Therefore, as in this study, the initial infection may have been influenced by smoking, or it may have occurred in older patients who smoke. Angiotensin-converting enzyme-2 expression in the lungs increases in smokers and patients with COPD (24).

Smoking can also affect the macrophage and cytokine responses and the body's ability to control infection. It has been suggested that smoking may affect interleukin 6 levels and influence inflammation and viral disease severity (25). Smoking is a risk factor for the acute and chronic severity of chikungunya virus infection (26). Similarly, the risk of infection with *Streptococcus pneumoniae*, Legionella, and *Mycoplasma pneumoniae* is 3–5 times higher among smokers (27). Therefore, it may affect the symptoms and severity of COVID-19 reinfection. It is unclear whether all reinfections, including symptomatic and non-symptomatic, are affected; however, symptomatic reinfections may be affected. Moreover, cigarette and e-cigarette users have increased adherence and colony formation of *Streptococcus pneumoniae* because of the upregulation of its receptor molecule (27). Nicotine may promote the cellular uptake mechanism of SARS-CoV-2 by nicotine-containing α7-nicotinic acetylcholine receptor signaling (28). The concomitant use of tobacco and e-cigarettes also increases the risk of being positive (29). The difference between the mechanism in current and former smokers is unknown; nevertheless, our results suggest that underlying disease and age play a role. It is also possible that the COVID-19 pandemic may have increased the number of smokers who were concerned or fearful of the effects of smoking (30). In addition, there have been behavioral changes regarding attempts to quit smoking (31), which may have increased the number of former smokers. Unfortunately, the results of this study suggest that quitting smoking does not immediately lead to a lower risk of reinfection. Therefore, the increased risk among patients who quit smoking could be because the number of underlying diseases, such as hypertension and diabetes, was higher in the former smoking group than in the non-smoking group, making it seem as if smoking cessation increased the risk of reinfection.

The percentage of reinfected patients was 3.7% in this study; we had difficulty interpreting the reinfection data. Our reported reinfection rate was <1% (32–34), and the mortality rate due to reinfection is considered low (35). The initial reinfection rate was low in this study, and the rate of reinfection in the initial 8 months was 0.36%, which was not different from that reported in other studies. This could have been because of the early stage of the epidemic and the short observation period. Subsequently, the number of reinfected patients increased sharply around the second quarter of 2021. Slezak et al. reported that more patients were hospitalized for suspected reinfection (36/315, 11.4%) than for initial infection (4,094/75,149; 5.4%) (36). It can be observed that the number of patients who were reinfected increased with a prolonged observation period. After July 2021, the delta variant predominated, and the risk of reinfection may have been higher than that reported previously (37).

Furthermore, the timing of this event coincided with an increase in the number of reinfection cases in our study. Reinfection occurs 6–9 months after the initial infection. However, it has been reported that reinfection was not observed at 12 months or later (11), and the decrease in anti-SARS-CoV-2 S-RBD IgG antibody in the BNT162b2 mRNA vaccine was approximately one-tenth of that at 6 months (38). The humoral and cellular immune responses are quantitatively lower, and the duration of protection is shorter in older people who commonly need nursing care than in healthy people. In this study, old age was the most relevant factor for reinfection. As for the persistence of antigens and RNA, the clearance of the virus is reduced in older people (over 65 years of age) (39), which may indirectly contribute to the risk of severe disease due to prolonged hospitalization and worsening of other basic diseases, making the occurrence of reinfection easier. In addition, antibody titers decrease in older people; however, the neutralizing antibody titers rise again with reinfection (10). In contrast, antibodies in healthcare workers have been reported to drop by only approximately half in 6 months (40). However, the minor decrease in antibody titers may be related to the maintenance of antibody titers, as reports of non-clinical cases were excluded (41). Healthcare workers have a lower risk of reinfection due to the maintenance of antibody titers (42).

The reduced risk of reinfection with vaccination is the most important confounding factor in this study. During the observation period of this study, vaccination coverage in the United States adult population was ~70% for the entire population, with more than 99% coverage achieved in those older than 65 years (43). However, it has been reported that unvaccinated individuals are 2.34 times more likely to be reinfected than vaccinated individuals (44).

Finally, the results of this study do not indicate that smoking lowers the risk of reinfection. Rather, the fact that one quits smoking due to a health condition that worsens may be used as a warning to increase the risk of reinfection.

This study had some limitations. First, the reinfection rate in this study was calculated based on a limited cohort; therefore, it should be interpreted with caution. Second, since only reinfection with the diagnosis was analyzed in this study, non-symptomatic reinfection that was not registered in the database was excluded. Third, investigating the impact of vaccination was necessary to improve the accuracy of the data, but the missing information hindered the identification of consciously unvaccinated individuals. However, the sample size was small. Fourth, vaccination was performed rapidly at venues and pharmacies, making it difficult to link the information in this database. Fifth, there were no data regarding the neutralizing antibody titers in the reinfected patients. Therefore, further research is needed to determine whether the presence of basic diseases directly affects the amount and half-life of neutralizing antibodies. Finally, we examined the relationship between the number of basic diseases, smoking status, and age with reinfection; however, the results did not reflect the weighting of each basic disease or the actual respiratory function of the patient.

In conclusion, former smokers who were older may have been exposed to factors that increase the potential risk of COVID-19 reinfection.

## Data availability statement

The raw data supporting the conclusions of this article will be made available by the authors, without undue reservation.

## Ethics statement

Ethical review and approval was not required for the study on human participants in accordance with the local legislation and institutional requirements. Written informed consent for participation was not required for this study in accordance with the national legislation and the institutional requirements.

## Author contributions

WA, TH, and MJ contributed to the draft, data curation, conceptualization, and initial design. WA and TH performed the formal analysis. WA, TH, and TU contributed to writing the manuscript. KA and HH contributed a critical review. KO contributed to all project decision-making. All authors contributed to the article and approved the submitted version.

## Conflict of interest

The authors declare that the research was conducted in the absence of any commercial or financial relationships that could be construed as a potential conflict of interest.

## Publisher's note

All claims expressed in this article are solely those of the authors and do not necessarily represent those of their affiliated organizations, or those of the publisher, the editors and the reviewers. Any product that may be evaluated in this article, or claim that may be made by its manufacturer, is not guaranteed or endorsed by the publisher.

## Abbreviations

CI: Confidence interval
COPD: Chronic obstructive pulmonary disease
EHR: Electronic health records
HR: Hazard ratios
HT: Hypertension
IP: Interstitial pneumonia
IQR: Interquartile range
SD: Standard deviation.
