# Supplementary material for: Smoking cessation in the elderly as a sign of susceptibility to symptomatic COVID-19 reinfection in the United States
Source: Front Public Health. 2022 Nov 22;10:985494. doi: 10.3389/fpubh.2022.985494 (PMC9733529; doi:10.3389/fpubh.2022.985494)
Supplement: Supplementary file 1 [file Table_1.DOCX]

Supplementary Material

Supplementary Table S1**.** Number of patients with coronavirus disease and reinfection in four regions

|  | Total infection, n | Reinfection, n (%) |
| --- | --- | --- |
| Northeast | 19,044 | 858 (4.5) |
| Midwest | 10,331 | 280 (2.7) |
| South | 96,674 | 3,966 (4.0) |
| West | 39,159 | 1,136 (2.9) |
| Unidentified | 112 | 0 (0) |
| Total | 165,320 | 6,133 (3.7) |

The number of patients per region was divided into four categories according to the US census classification (Northeast, Midwest, South, and West)

**Supplementary S2.** Analysis of reinfection based on vaccination data

|  | No vaccination data | First vaccination completed | Second vaccination completed | Total |
| --- | --- | --- | --- | --- |
| Initial infection, n | 153,679 | 1,333 | 4,175 | 159,187 |
| Reinfection, n | 5,924 | 52 | 157 | 6,133 |
| Reinfection ratio, % | 3.7 | 3.8 | 3.6 | 3.7 |
